# Synchrotron MicroCT Reveals the Potential of the Dual Contrast Technique for Quantitative Assessment of Human Articular Cartilage Composition

**DOI:** 10.1002/jor.24479

**Published:** 2019-10-14

**Authors:** Miitu K. M. Honkanen, Annina E. A. Saukko, Mikael J. Turunen, Rubina Shaikh, Mithilesh Prakash, Goran Lovric, Antti Joukainen, Heikki Kröger, Mark W. Grinstaff, Juha Töyräs

**Affiliations:** ^1^ Department of Applied Physics University of Eastern Finland Kuopio Finland; ^2^ Diagnostic Imaging Center Kuopio University Hospital Kuopio Finland; ^3^ Department of Medical Physics Turku University Hospital Turku Finland; ^4^ SIB Labs University of Eastern Finland Kuopio Finland; ^5^ A.I. Virtanen Institute for Molecular Sciences University of Eastern Finland Kuopio Finland; ^6^ Centre d'lmagerie BioMédicale École Polytechnique Fédérale de Lausanne Lausanne Switzerland; ^7^ Swiss Light Source Paul Scherrer Institute Villigen Switzerland; ^8^ Department of Orthopedics, Traumatology and Hand Surgery Kuopio University Hospital Kuopio Finland; ^9^ Departments of Biomedical Engineering, Chemistry, and Medicine Boston University Boston Massachusetts; ^10^ School of Information Technology and Electrical Engineering The University of Queensland Brisbane Queensland Australia

**Keywords:** dual contrast agent, CA4+, cationic contrast agent, contrast‐enhanced computed tomography, CECT

## Abstract

Dual contrast micro computed tomography (CT) shows potential for detecting articular cartilage degeneration. However, the performance of conventional CT systems is limited by beam hardening, low image resolution (full‐body CT), and long acquisition times (conventional microCT). Therefore, to reveal the full potential of the dual contrast technique for imaging cartilage composition we employ the technique using synchrotron microCT. We hypothesize that the above‐mentioned limitations are overcome with synchrotron microCT utilizing monochromatic X‐ray beam and fast image acquisition. Human osteochondral samples (*n* = 41, four cadavers) were immersed in a contrast agent solution containing two agents (cationic CA4+ and non‐ionic gadoteridol) and imaged with synchrotron microCT at an early diffusion time point (2 h) and at diffusion equilibrium (72 h) using two monochromatic X‐ray energies (32 and 34 keV). The dual contrast technique enabled simultaneous determination of CA4+ (i.e., proteoglycan content) and gadoteridol (i.e., water content) partitions within cartilage. Cartilage proteoglycan content and biomechanical properties correlated significantly (0.327 < *r* < 0.736, *p* < 0.05) with CA4+ partition in superficial and middle zones at both diffusion time points. Normalization of the CA4+ partition with gadoteridol partition within the cartilage significantly (*p* < 0.05) improved the detection sensitivity for human osteoarthritic cartilage proteoglycan content, biomechanical properties, and overall condition (Mankin, Osteoarthritis Research Society International, and International Cartilage Repair Society grading systems). The dual energy technique combined with the dual contrast agent enables assessment of human articular cartilage proteoglycan content and biomechanical properties based on CA4+ partition determined using synchrotron microCT. Additionally, the dual contrast technique is not limited by the beam hardening artifact of conventional CT systems. © 2019 The Authors. *Journal of Orthopaedic Research*® published by Wiley Periodicals, Inc. on behalf of Orthopaedic Research Society. J Orthop Res 38:563–573, 2020

Cartilage injury can initiate the development of post‐traumatic osteoarthritis (PTOA) if not detected and treated early after injury. Therefore, early diagnosis is crucial for the initiation of surgical or pharmaceutical treatment to slow down the lesion progression to PTOA.^1^, ^2^, ^3^, ^4^ Currently available clinical imaging modalities to assess cartilage condition (proteoglycan [PG] distribution, collagen network organization, and water content) include magnetic resonance imaging (MRI) and computed tomography (CT).^5^ However, the usability of MRI in diagnostics is limited by availability, relative long acquisition time, and incompatibility with metallic implants.^6^ In addition, CT without contrast enhancement is capable of detecting only joint space narrowing and changes in bony structures at late‐stage of the disease.^6^ Thus, new sensitive and quantitative imaging methods are needed for the detection of minor articular cartilage lesions and early osteoarthritic degeneration.

Contrast‐enhanced computed tomography (CECT) enables detection of changes in cartilage composition along with quantitative evaluation of subchondral bone density and structure.^7^, ^8^, ^9^, ^10^, ^11^ Advantages of CECT compared with MRI are shorter acquisition times, higher resolution, and lower costs. Delayed CECT^7^, ^8^, ^12^ allows the detection of early degenerative changes since degeneration alters contrast agent diffusion within cartilage. Originally, anionic contrast agents were used in CECT^13^, ^14^, ^15^; however, recent studies have introduced cationic contrast agents for cartilage imaging showing improved sensitivity for PG loss.^16^, ^17^ Cationic contrast agents are more sensitive to cartilage PG content at diffusion equilibrium than anionic agents due to electrostatic attraction between cationic molecules and negatively charged PGs. However, cationic contrast agents have limitations in assessing PG content of cartilage at clinically feasible early diffusion time points, that is, 1–2 h after contrast agent administration.^18^ At early diffusion time points, contrast agent diffusion into cartilage is affected by water content and surface permeability as well as by the PG content, diminishing the sensitivity of cationic contrast agents to variations in PG content. As a solution, we introduced a dual contrast agent (a mixture of two contrast agents) and a dual energy CT technique (two X‐ray energies) for imaging at diffusion equilibrium.^19^ In that study, we used the dual contrast agent composed of cationic, iodine‐based (CA4+,^20^, ^21^ *q *= +4, and iodine *k*‐edge 33.2 keV) and non‐ionic, gadolinium‐based (gadoteridol, *q *= 0, and gadolinium *k*‐edge 50.3 keV) contrast agents. The diffusion of non‐ionic contrast agent reflects the water content and permeability when the contrast agent partition is determined prior to diffusion equilibrium. Further, by normalizing the cationic contrast agent distribution with that of non‐ionic agent distribution, the PG content within cartilage is estimated more accurately.^19^, ^22^ This technique is based on exploiting two X‐ray energies that enable simultaneous determination of the distributions of the contrast agents within cartilage. To achieve this, X‐ray energies must be chosen carefully based on the applied contrast agents and their element‐specific *k*‐edges (i.e., photoelectric absorption edges).

The main limitation of conventional microCT and clinical CT scanners is the relatively wide energy spectrum. With one tube voltage, the photon energies can spread on both sides of the *k*‐edge of the chosen contrast agent. In addition, beam hardening artifacts can occur. These factors might limit the accuracy of the dual contrast technique. Moreover, with conventional microCT scanners, high‐resolution images require long acquisition time. When imaging during active diffusion (i.e., early diffusion time points), the image acquisitions with both energies should, in theory, be conducted simultaneously. To achieve very high resolution with relatively short imaging time (~ 2 min per acquisition), synchrotron‐based microCT^23^ can be used to validate the technique. As monochromatized synchrotron microCT X‐ray beams exhibit a narrow energy spectra, the contrast difference between the two images and between the contrast agents is maximized.

Herein, for the first time, we employ two different monochromatic X‐ray energies in high‐resolution synchrotron‐based microCT to image the diffusion of dual contrast agent in human articular cartilage. The aim of this study is to assess the full potential of the dual contrast technique to detect human articular cartilage degeneration at a clinically relevant diffusion time point (2 h) and at diffusion equilibrium (72 h). We hypothesize that synchrotron‐based microCT will provide sensitive dual contrast diagnosis of spontaneous arthritic human articular cartilage and that limitations of conventional CT systems are overcome with synchrotron microCT employing monochromatic X‐ray beam and fast image acquisition.

## METHODS

### Contrast Agent Preparation

The dual contrast agent was composed of CA4 + (5,5′‐(malonylbis(azanediyl))bis(N^1^,N^3^‐bis(2‐aminoethyl)‐2,4,6‐triiodoisophthalamide, *q *=*  *+4, *M *= 1,499.88 g/mol) and gadoteridol (Prohance®; Bracco International B. V., Amsterdam, Netherlands, *q *= 0, *M *= 558.69 g/mol) diluted with phosphate‐buffered saline (PBS). Two dual contrast agent immersion baths (for 2 and 72 h immersions) containing 5 mg I/ml and 10 mg Gd/ml were prepared. Inhibitors of proteolytic enzymes (5 mM ethyleneadiaminetetra‐acetic acid [EDTA; VWR International, Fontenay‐sous‐Bois, France] and 5 mM benzamidine hydrochloride hydrate [Sigma‐Aldrich Inc., St. Louis, MO]) were added to both immersion baths. Furthermore, penicillin–streptomycin–amphotericin B (antibiotic antimycotic solution: 100 units/ml penicillin, 100 µg/ml streptomycin, and 0.25 µg/ml amphotericin B, Sigma‐Aldrich Inc., St. Louis, MO) was added to the 72‐h immersion bath to minimize the sample degeneration. The osmolalities of the contrast agent mixtures were ~308 and ~297 mOsm/kg for the 2‐ and 72‐h immersion baths, respectively. The osmolalities of the contrast agents and PBS were measured with a freeze‐point osmometer (Halbmikro‐osmometer; GWB, Knauer & CO GmbH, Berlin, West‐Germany).

### Sample Preparation

**Figure 1:**
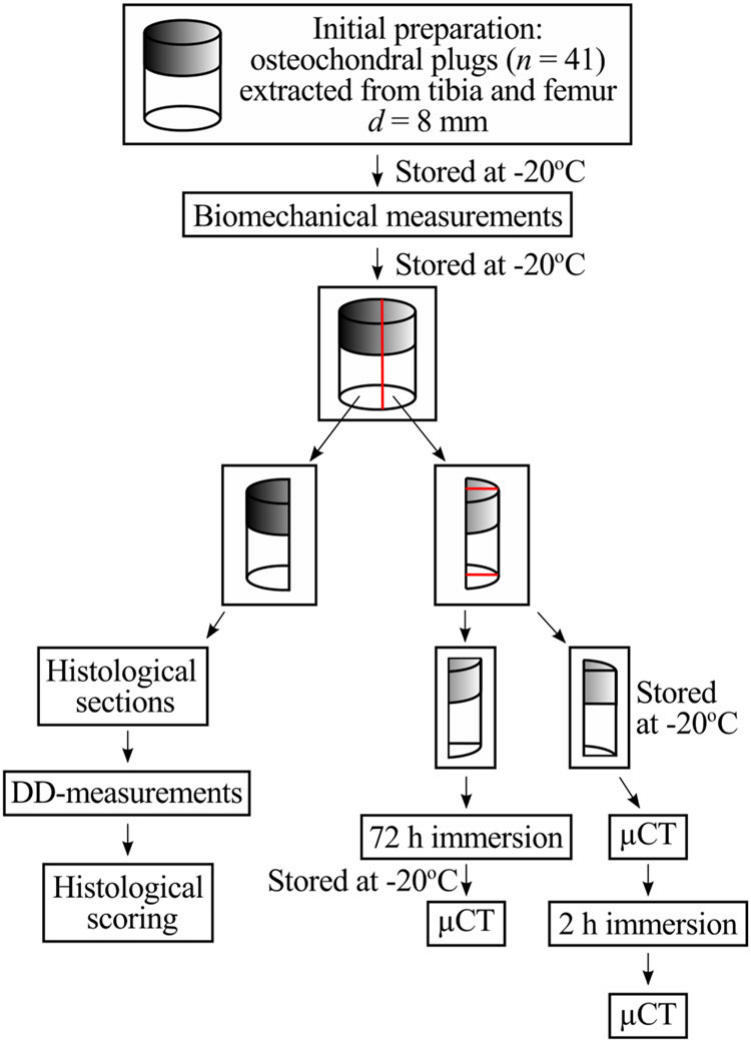
Study work‐flow and the osteochondral sample processing protocol [Color figure can be viewed at wileyonlinelibrary.com]

A total of 41 osteochondral samples (*d *= 8 mm, thickness = 2.21 ± 0.46 mm) were extracted from human cadavers’ (*N *= 4, mean age 71.3 [from 68 to 79]) left and right proximal tibia (*N = *8) and distal femur (*N = *8). The Research Committee of the Northern Savo Hospital District (Kuopio University Hospital, Kuopio, Finland, ethical permission number: 134/2015) approved the sample collection. A detailed flowchart describing sampling and experiments is shown in Fig. 1. The specimens were stored at −20°C until thawed for the biomechanical measurements. Subsequently, the samples were refrozen, stored at −20°C, and later halved. One half was sectioned for digital densitometry measurements and histological analysis, whereas the other half was halved again to quarters. The quarter sides were sealed carefully with cyanoacrylate (Super Glue Precision, Loctite, Düsseldorf, Germany) to ensure the contrast agent diffusion only through the articulating surface. The first quarter was immersed in PBS and the second quarter in the dual contrast agent mixture bath (4.4 ml, 100 times the largest cartilage volume) for 72 h at +8°C to avoid tissue degeneration. The bath was gently agitated using Gyro rocker (STR9 Gyro rocker Platform Rocker; Stuart Scientific, Staffordshire, UK) during the immersion. Afterwards, the samples were stored at −20°C until the synchrotron microCT measurements.

### Biomechanical Measurements

Biomechanical properties of cartilage (*n* = 40, data for one sample was lost due to technical malfunction) were determined by indentation loading using a custom‐made material tester equipped with an actuator having displacement resolution of 0.1 µm (PM500‐1 A; Newport, Irvine, CA) and a load cell with 5 mN force resolution (Sensotec, Columbus, OH).

First, the average cartilage thickness was calculated from four measurements around the cylindrical osteochondral sample using a vernier caliper (resolution = 0.01 mm). Subsequently, samples (from bone) were glued to the bottom of a sample holder, which was then filled with PBS to fully immerse the samples. The container was adjusted to set the cartilage surface in perpendicular contact with cylindrical, custom‐made, flat‐ended, metallic indenter (*d *= 728 µm [*n *= 7, the indenter was damaged after the first 7 samples were measured] or *d *= 667 µm [*n = *33]). After reaching mechanical equilibrium at pre‐stress of 12.5 kPa,^24^ three compressive steps (3 × 5% of uncompressed cartilage thickness with a ramp rate of 100%/s) were applied using 900 s relaxation time in between the compressive steps. The Hayes model^25^ was used to calculate the equilibrium modulus (fit to the last three equilibrium points) and instantaneous modulus (the ramp phase of the third step). The thickness of the samples was verified and corrected based on microCT images. The Poisson's ratios applied in the calculations were as follows: equilibrium modulus (*ν* **= **0.2)^26^ and instantaneous modulus (*ν* **= **0.5).^27^, ^28^

### Synchrotron‐Based MicroCT Imaging

The microCT imaging was conducted at X02DA TOMCAT beamline (Swiss Light Source, Paul Scherrer Institute, Villigen, Switzerland). Two monochromatic X‐ray energies (32 and 34 keV) were selected by utilizing a double‐multilayer monochromator (with a spectral bandwidth of about 2–3%) based on iodine *k*‐edge (33.2 keV). The image acquisition was conducted by combining 1:1 magnifying visible light optics microscope (Optique Peter, Lyon, France), a 300‐µm thick scintillator (LuAg, CRYTUR spol.s.r.o., Czech Republic), and an sCMOS detector (pco.Edge 5.5; PCO AG, Kelheim, Germany). A field of view (FOV) of 16.640 × 3.497 mm^2^ and an isometric voxel size of 6.5 × 6.5 × 6.5 µm^3^ were applied. To minimize the radiation exposure of the samples, an off‐beam alignment system was used.^29^

In order to estimate the dose deposited on each sample, the X‐ray flux was measured with calibrated passivated implanted planar silicon diodes^29^ and yielded 9.8 × 10^10^ photons/mm^2^/s for the 32 keV and 1.0 × 10^11^ photons/mm^2^/s for the 34 keV, respectively. The cartilage samples were then modeled as soft tissue (ICRU‐44) by considering the X‐ray mass energy‐absorption coefficient from the NIST database,^30^ which yielded doses of 0.47 and 0.43 Gy for the two energies (32 and 34 keV), respectively. These values, however, do not consider the presence of the contrast agents which would need to be taken into account when estimating the radiation dose deposition among different tissues (such as cartilage and bone).^31^, ^32^

**Figure 2:**
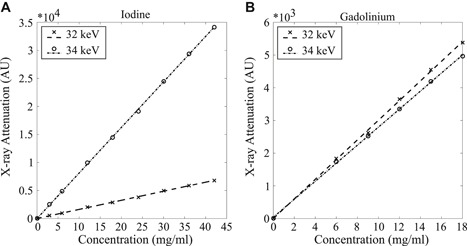
The contrast agent series for (A) CA4+ (iodine, I) with 3, 6, 12, 18, 24, 30, 36, and 42 mg I/ml and (B) gadoteridol (gadolinium, Gd) with 6, 9, 12, 15, and 18 mg Gd/ml were imaged with energies of 32 and 34 keV separately to determine the iodine and gadolinium mass attenuation coefficients. The figures show the X‐ray attenuation as a function of the true concentration of the contrast agents. The equations for X‐ray attenuation (AU) as a function of contrast agent concentration for iodine were *µ*_I,32keV_ = 162 ×* C*_I_ + 16 (*R* ^2^ = 0.999) and *µ*_I,34keV_ = 812 ×* C*_I_ + 12 (*R* ^2^ = 1.000); for gadolinium *µ*_Gd,32keV_ = 300 ×* C*_Gd_ + 1 (*R* ^2^ = 0.999) and *µ*_Gd,34keV_ = 276 ×* C*_Gd_ + 39 (*R* ^2^ = 1.000).

First, a set of contrast agent phantoms with varying CA4+ and gadoteridol concentrations in distilled water were imaged using both energies. In the phantoms, the iodine (CA4+) concentrations were 3, 6, 12, 18, 24, 30, 36, and 42 mg I/ml and the gadolinium (gadoteridol) concentrations were 6, 9, 12, 15, and 18 mg Gd/ml (Fig. 2). In addition, eight mixture phantoms with iodine/gadolinium concentrations of 3/18, 6/16, 10/14, 16/12, 20/10, 26/8, 32/6, and 40/3 mg/ml were imaged.

**Figure 3:**
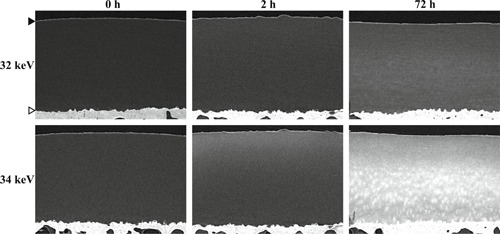
Tomographic slices of the computed tomography (CT)‐reconstructed volume acquired with 32 and 34 keV photon energies at different time points (0, 2, and 72 h) after the contrast agent immersion. The articulating surface and cartilage–bone interface are marked with black and white arrowheads, respectively. The contrast agent mainly responsible for inducing the contrast is CA4+ and gadoteridol for 34 and 32 keV images, respectively

Prior to the immersion in dual contrast agent mixture, the non‐contrast images (Fig. 3) of the osteochondral samples were acquired by measuring the samples in the air. Next, the samples were immersed in contrast agent mixture bath (4.4 ml, ≥100 times the cartilage volume) for 2 h at +7°C. Further, the sample quarters immersed for 72 h in contrast agent mixture before storage (−20°C) were after thawing immersed again for 1 h to ensure the diffusion equilibrium. After the immersions, the image acquisition (Fig. 3) was conducted as follows, first with 34 keV X‐ray beam, then with 32 keV, and finally again with 34 keV. Two acquisitions for 34 keV were conducted and averaged to minimize the time difference between acquisitions with the two energies. The imaging time was minimized to be approximately 129 s per acquisition with the minimal time difference between the subsequent acquisitions (~5 min 31 s between the starting times of the scans).

### Data Analysis

The concentrations in the contrast agent mixture phantoms and the contrast agent partitions within cartilage were solved using Beer–Lambert law and Bragg's additive rule for mixtures,^33^ as were done in our earlier studies^19^, ^22^, ^34^:

(1) $$\alpha_{E}=\mu_{I,E}C_{I}+\mu_{\text{Gd},E}C_{\text{Gd}}$$

where *α* is the total X‐ray attenuation of the contrast agent mixture within phantom or cartilage with energy *E*, *µ* is the mass attenuation coefficient, and *C* is the concentration of iodine (I) or gadolinium (Gd) in the mixture. The contrast agent concentrations in the mixtures and within cartilage are solved using Equation (1) based on the radiation intensities of two different X‐ray energies applied (in this study 32 and 34 keV) as follows:

(2) $$C_{I}=\frac{\mu_{\text{Gd},32}\alpha_{34}-\mu_{\text{Gd},34}\alpha_{32}}{\mu_{I,34}\mu_{\text{Gd},32}-\mu_{I,32}\mu_{\text{Gd},34}}$$

(3) $$C_{\text{Gd}}=\frac{\mu_{I,34}\alpha_{32}-\mu_{I,32}\alpha_{34}}{\mu_{I,34}\mu_{\text{Gd},32}-\mu_{I,32}\mu_{\text{Gd},34}}$$

The articulating surface and bone‐cartilage interface were manually determined using a segmentation software (Seg3D, version 2.2.1, 2015; University of Utah, Salt Lake City, UT) for every sample at both time points. Due to the limited vertical FOV of approximately 3.5 mm, the cartilage–bone interface was not visible for 9/41 samples, and therefore, for those samples the interface was determined to be at the image border, resulting in the loss of the calcified cartilage (<200 µm). The volume of interest (VOI, a cylinder with the diameter of 1,950 µm and height of cartilage thickness) was selected from the center of the osteochondral sample by using a custom‐made MATLAB (R2015b and R2017b; The MathWorks, Inc., Natick, MA) code.^35^ The 34‐keV acquisitions conducted at the beginning and end of the imaging sequence were averaged to minimize the error caused by ongoing diffusion.

**Figure 4:**
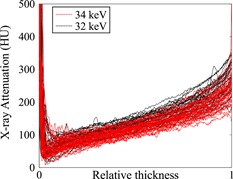
Non‐contrast (baseline) X‐ray attenuation profiles of cartilage samples acquired with X‐ray energies of 32 and 34 keV. In horizontal axis, 0 refers to articular surface and 1 to cartilage–bone interface [Color figure can be viewed at wileyonlinelibrary.com]

First, a non‐contrast‐enhanced X‐ray attenuation profile (horizontal average of VOI, Fig. 4), attained from the halves that initially contained no contrast agent, was subtracted from the X‐ray attenuation profiles of samples with diffused contrast agents. Next, the concentration profiles for CA4 + and gadoteridol were determined (Equations (2) and (3)) and from those, the partition profiles were calculated as follows:

(4) $$P\text{artition}=\frac{C_{\text{cartilage}}}{C_{\text{bath}}}$$

For further analyses, the cartilage thickness was divided into superficial (0–10%), middle (10–40%), deep (40–100%) zones, and into full cartilage thickness (0–100%) starting from articulating surface (0%) to cartilage–bone interface (100%). The decrease in bath concentration (due to the contrast agent diffusion into cartilage) was taken into account when calculating the partition profiles. The masses of gadolinium and iodine were calculated based on their contents within cartilage and sample volume and then deduced from the masses of gadolinium and iodine in the bath. With this information, the bath concentrations of CA4+ and gadoteridol at 2‐ and 72‐h time points were determined. Finally, the partitions of CA4+ and gadoteridol were calculated using Equation (4). Further, CA4+ partition profiles were normalized (i.e., divided) with those of gadoteridol profiles to enhance the accuracy of detecting the PG content within the sample.

### Digital Densitometry and Histological Grading

The sample halves were decalcified in EDTA, cut into 3 µm thick sections, and stained with Safranin‐O. Safranin‐O is a cationic dye, and it is stoichiometrically attracted to PGs, thus quantitatively revealing the PG distribution within cartilage.^36^ The optical density (OD, i.e., PG content) was determined with digital densitometry measurements using a light microscope (Nikon Microphot‐FXA; Nikon Co., Tokyo, Japan) with a monochromatic light source (wavelength 492 ± 8 nm) and a 12‐bit CCD camera (ORCA‐ER; Hamamatsu Photonics K.K., Hamamatsu, Japan). Three sections per sample were averaged, and the calibration was conducted using neutral density filters ranging from 0 to 3.0.

The severity of OA was evaluated along three histological grading systems: Mankin score,^37^ Osteoarthritis Research Society International (OARSI) grading,^38^ and International Cartilage Repair Society (ICRS) grading.^39^ For Mankin grading system, abnormalities in structure (from 0 to 6 points), cellularity (0–3), Safranin‐O staining (0–4), and tidemark integrity (0–1) were evaluated. In OARSI grading the lesion depth, size, and cartilage condition were evaluated (points from 0 to 6). ICRS grade (0–4) was set based on the lesion depth. Randomly ordered and blind‐coded sections were scored by four assessors (M. Honkanen, N. Hänninen, M. Prakash, and R. Shaikh). Finally, the scores of the three sections per sample were averaged. Same sections were utilized for DD and histological grading.

### Statistical Analysis

The relations between the measured and the true contrast agent concentrations in the mixture phantoms were evaluated with Pearson's correlation analysis. The statistical dependency between contrast agent partitions within cartilage and biomechanical data (equilibrium and instant modules), and PG content (OD) were assessed with Pearson's correlation, and dependency between contrast agent partitions and histological score and gradings (Mankin score, ICRS grading, and OARSI grading) were assessed with Spearman's correlation. The improvement in correlation by the normalization of the CA4+ partition with that of gadoteridol was tested according to Steiger^40^ for Pearson's correlations and with bootstrapping for Spearman's correlations. All the statistical analyses were conducted using SPSS (v. 25.0; SPSS Inc., IBM Company, Armonk, NY).

## RESULTS

The microCT measured composition of the contrast agent mixture phantoms agreed with the true composition (Pearson's correlation, *R* ^2^ = 1.000, *p *< 0.0001). The difference between the true and measured concentrations were 1.46 ± 0.37% for iodine and 2.62 ± 1.11% for gadolinium (Table 1).

**Table 1:** The True and the Measured Contrast Agent (Iodine/Gadolinium) Concentrations and Their Errors in the Mixture Phantoms

True (mg/ml)	Measured (mg/ml)	Error (%)
3/18	3.04/17.63	1.17/2.06
6/16	5.90/15.65	1.68/2.20
10/14	9.83/13.50	1.74/3.56
16/12	16.09/11.76	0.58/1.97
20/10	19.69/9.56	1.54/4.37
26/8	25.53/8.02	1.82/0.31
32/6	31.43/5.80	1.79/3.39
40/3	39.44/3.09	1.40/3.07

**Figure 5:**
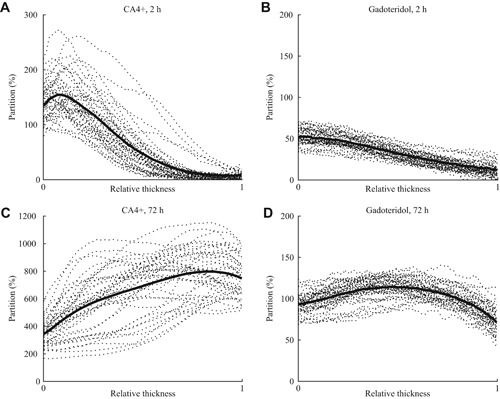
Contrast agent partitions (*n *= 41, dashed lines) and the mean partition (thick line) as a function of the cartilage relative thickness for (A) CA4+ and (B) gadoteridol at 2‐h diffusion time point and, (C) CA4+ and (D) gadoteridol at 72 h after diffusion. In the horizontal axis, 0 refers to the articular surface and 1 refers to cartilage–bone interface.

The contrast agent partition profiles within cartilage for CA4+ and gadoteridol at 2‐ and 72‐h diffusion time points are presented in Fig. 5. At the 2‐h diffusion time point, CA4+ and gadoteridol partition showed a decreasing trend as a function of cartilage thickness and had mean full thickness partitions of 66.1 ± 21.9% and 32.8 ± 6.2%, respectively. After 72 h of diffusion the CA4+ and gadoteridol partitions showed increasing and decreasing trends, respectively, toward the deep cartilage and had mean full thickness partitions of 658.7 ± 155.2% for CA4+ and 103.4 ± 9.9% for gadoteridol. The 2‐h CA4+ partition positively correlated with increasing PG content (i.e., OD, 0.463 < *r < *0.736, 0.000 < *p *< 0.002, Table 2) in superficial and middle zones (0–10 and 10–40%). A significant negative correlation (*r*_S_ = −0.570, *p *< 0.000, Table 3) was found between the superficial CA4+ partition and Mankin score at the 2‐h time point. In addition, the 2‐h CA4+ partition in superficial and middle zones, and in full cartilage correlated significantly with equilibrium modulus (0.331 < *r *< 0.659, 0.000 < *p *< 0.037) and instantaneous modulus (0.378 < *r *< 0.511, 0.001 < *p *< 0.016, Table 2). At the 2‐h time point, gadoteridol partition in superficial and middle zones and in full cartilage negatively correlated to equilibrium modulus (−0.499 < *r *< −0.389, 0.001 < *p *< 0.013) and instantaneous modulus (−0.376 < *r *< −0.361, 0.017 < *p *< 0.022, except in full cartilage thickness). Further, 2‐h gadoteridol partition in all zones and in full cartilage correlated significantly with Mankin score (0.334 < *r*_S _<_ _0.651, 0.000 < *p* < 0.033), OARSI score (0.322 < *r*_S_ < 0.659, 0.000 < *p *< 0.040), and ICRS score (0.328 < *r*_S_ < 0.643, 0.000 < *p *< 0.037). At diffusion equilibrium CA4+ partition correlated significantly (0.685 < *r *< 0.766, *p *< 0.0001) with PG content in full cartilage and all zones. Importantly, at 2‐h time point, the Spearman's correlation coefficients were significantly (*p *< 0.05) higher between the normalized CA4+ partitions in the superficial and middle zones, and in full cartilage thickness and histological score and grades (Mankin, OARSI, and ICRS) than for non‐normalized CA4+ partitions (Table 3). Furthermore, at 2‐h time point, the Pearson correlation coefficients were significantly (*p *< 0.05) higher between the normalized CA4+ partitions in the middle zone and in full cartilage thickness and optical density, equilibrium, and instantaneous modules than for non‐normalized CA4+ partitions (except for OD in full thickness cartilage; Table 2). At 72‐h time point significant (0.366 <* r *< 0.824, 0.000 < *p *< 0.020) correlations were found between normalized CA4+ partition and reference parameters (OD, equilibrium and instant moduli, and Mankin score) in the superficial and middle layers, and in full thickness cartilage. The normalization significantly improved the correlation between CA4+ partition and the reference parameters in superficial (OD, equilibrium and instant moduli, OARSI, and ICRS), middle (equilibrium and instant moduli, OARSI, and ICRS), deep (equilibrium modulus) zones, and full thickness cartilage (equilibrium and instant moduli).

**Table 2:** Pearson's Correlation Coefficients for CA4+, Normalized CA4+ (CA4+ norm), and Gadoteridol Partitions When Compared With Optical Density (Proteoglycan Content), and Biomechanical Properties (Equilibrium and Instant Moduli) of Cartilage

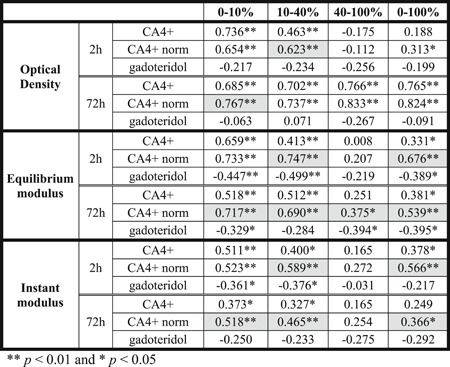

Significantly (*p *< 0.05) higher correlations for normalized CA4+ partition  than for non‐normalized CA4+ partition are highlighted with light gray.

**Table 3:** Spearman's Correlation Coefficients for CA4+, Normalized CA4+ (CA4+ norm), and Gadoteridol When Compared With Mankin Score, Osteoarthritis Research Society International (OARSI) Grading and International Cartilage Repair Society (ICRS) Grading

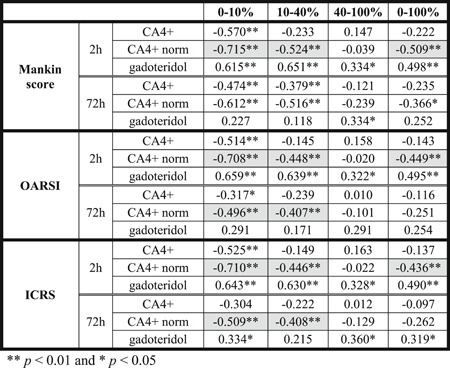

Significantly (*p *< 0.05) higher correlations for normalized CA4+ partition  than for non‐normalized CA4+ partition are highlighted with light gray.

## DISCUSSION

As compared with anionic contrast agents, cationic agents provide better contrast and are more sensitive to variation in PG content.^17^ However, the clinical potential of cationic contrast agent (CA4+) suffers from different degeneration‐related factors having opposite effects on CA4+ diffusion. At early diffusion time points, degeneration‐induced PG loss decreases the diffusion while the increase in water content and permeability increase the diffusion. The dual contrast technique overcomes this limitation and enhances the detection of PG content in human cartilage at diffusion equilibrium^19^ as well as at early diffusion time points in bovine^34^ and in human^22^ cartilage. In this study, we have shown that dual contrast‐enhanced synchrotron microCT enables detection of the decreased PG content in osteoarthritic human articular cartilage. Furthermore, the normalization of the CA4+ partition with the gadoteridol partition significantly (*p < *0.05) improved the correlations with the reference parameters at an early diffusion time point and at diffusion equilibrium.

The measured CA4+ and gadoteridol concentrations in the mixture phantoms correlate linearly with the true concentrations (*R* ^2^ = 1.000, *p < *0.0001). This correlation along with minor error between the true and measured concentrations, reflects that the determination of the concentrations is accurate and linear within whole range of concentrations with the present dual contrast technique. A similar correlation between measured and true concentrations in the mixture phantoms was observed in our recent synchrotron microCT study with different X‐ray energies (25 and 37 keV).^34^ In the present study, we took advantage of the narrow X‐ray beam energy bandwidth and used the image acquisition energies (32 and 34 keV) very close to iodine *k*‐edge (33.2 keV) to minimize the time difference between the measurements due to energy change and to maximize the image contrast.

CA4+ distributions within cartilage were as expected (at 2 h decreasing and at 72 h increasing trends toward cartilage–bone interface) and in line with the previous studies at both diffusion time points measured in human^19^, ^22^ and bovine articular cartilage.^34^ For gadoteridol, the shapes of the distribution profiles were similar to those reported earlier at both diffusion time points.^19^, ^22^, ^34^ However, the gadoteridol partitions (over 100%) at the diffusion equilibrium were unexpectedly high (Fig. 5D) as gadoteridol should diffuse into cartilage according to cartilage water content (which is around 80%^41^). Moreover, the CA4+ partitions were slightly higher than those reported earlier^19^, ^22^ even though the sample preparation and experimental conditions were similar. Beam hardening cannot explain the high gadoteridol partitions as a monochromatic beam was used. The applied relatively high radiation doses could lead to ionization of the contrast agent and cartilage matrix. However, the image acquisition time was short (around 2 min), and the imaging was conducted in air. In addition, the same samples were imaged only once (72 h) or twice (baseline and 2 h), so the high gadoteridol partition was likely not due to ionization. Possibly, the very high diffusion flux of CA4+ may have increased the diffusion of the gadoteridol. We have observed this phenomenon also in another (unpublished) study where diffusion of the dual contrast agent as a time series was investigated using a conventional microCT system. However, this is purely speculation at this moment and requires further investigation. A similar high gadoteridol partition has been observed also by Gillis et al.^42^

The correlations between CA4+ partition in the superficial zone and reference parameters were significant (*p *< 0.05) at 2 h after contrast agent immersion. In general, the correlation coefficients between CA4+ partition and all the reference parameters (PG content, biomechanical properties, Mankin score, OARSI, and ICRS grades, Tables 2 and 3) were significantly (*p *< 0.05) greater when the CA4+ partition was normalized with that of gadoteridol in the superficial and middle zones, and in full cartilage thickness at both diffusion time points. At the 2‐hour time point, the diffusion profiles show minimal CA4+ and gadoteridol accumulation in the deep cartilage (Fig. 5). This explains the lack of correlations between the contrast agent partitions in the deep cartilage and reference parameters. At early time points, diffusion of CA4+ is controlled by fixed charge density, permeability and water content, while diffusion of gadoteridol depends strongly on permeability and water content. Therefore, the normalization improves the discrimination between the healthy and degenerated cartilage tissue at an early diffusion time point, which agrees with the literature.^22^, ^34^ At diffusion equilibrium, the very high gadoteridol partition (over 100%) affects the accuracy of the normalization, and therefore, explains the lack of statistically significant difference between the correlations with the overall condition (Mankin score) and PG content of non‐normalized and normalized CA4+ partitions.

Several limitations are present in this synchrotron microCT study with human osteochondral samples. First, the theory behind the dual contrast technique assumes that the images are acquired simultaneously with two separate energies. This was not possible in the present study. However, the time difference between the 32 and 34 keV acquisitions was approximately only 5 min 31 s. Furthermore, the 34 keV acquisitions conducted at the beginning and end of the imaging sequence were averaged to more closely match the 32 and 34 keV acquisitions. This was especially necessary for the 2‐h time point when the diffusion is very fast. After averaging, the time difference between the 32 and 34 keV acquisitions was minimal and the effect to the results is assumed to be negligible. The FOV height (~3.5 mm) was close to the thickness of the thickest (~3.2 mm) cartilage samples. To ensure the visibility of the cartilage surface, a safety margin was left at the top of the image. Therefore, the cartilage–bone interface was invisible for 9/41 samples, and a region of maximum 200 µm of the deepest cartilage was lost, resulting in a minor shift of the zones and therefore, a minor error in the contrast agent partition in every zone for those samples. Another limitation of this study was the low number of cadavers (*n *= 4; eight knee joints). As from two to eight osteochondral samples were extracted from the same knee joint, there is a dependency between the samples. In addition, the samples were extracted from tibia and femur as well as both medial and lateral sides of the joint. This causes degeneration independent variation in cartilage composition, which cannot be differentiated with scoring and grading systems and therefore, might have led to weaker correlations. Furthermore, the samples were frozen and thawed three times before the synchrotron microCT experiment, which may have caused some degeneration. However, this degeneration can be assumed to be similar between the samples and with no major effect on biomechanical properties.^43^, ^44^, ^45^

In vivo, the diffusion of an anionic contrast agent reaches its maximum concentration in the cartilage at 30–60 min after the injection^7^ being a result of physiological clearance, while it takes longer for cationic contrast agents (hours; currently the only in vivo report is with rabbit knee joints).^46^ The diffusion time and in vitro conditions of the contrast agents are limitations of this laboratory study and for the clinical applicability. However, for post‐mortem analysis, ex vivo studies, and pre‐clinical in vitro and in vivo studies this is less of a concern. The primary focus of this laboratory study was to evaluate the potential of the dual contrast technique for imaging cartilage composition using a synchrotron microCT. The next step toward clinical setting is an extensive time series with ex vivo measurements (cadaveric knee joints) at early diffusion time points to refine the method and to explore alternative strategies to increase the diffusion rate.

To conclude, the present results show that the dual energy technique combined with the dual contrast agent enables determination of overall condition, equilibrium and instantaneous moduli, and water and PG contents in human osteoarthritic cartilage at a clinically relevant diffusion time point (2 h). This synchrotron microCT study reveals the potential of the dual contrast technique and provides the proof‐of‐concept for assessment of cartilage composition, as imaging‐related uncertainties were minimized. The results are in line with our earlier studies with conventional CT systems and indicate that beam hardening error is minimal with conventional CT scanners when imaging in vitro samples. The dual contrast technique offers significant diagnostic potential for detection of articular cartilage degeneration.

## AUTHORS’ CONTRIBUTION

M.K.M.H., A.E.A.S., M.J.T., G.L., and J.T. designed the study. H.K. and A.J. applied for ethical approval and participated in sample collection. CA4 +  contrast agent was synthesized in the laboratory of M.W.G. Synchrotron microCT measurements were conducted by M.K.M.H., A.E.A.S., M.J.T., G.L., and J.T. M.K.M.H., A.E.A.S., M.J.T., and J.T. participated in the data interpretation. Biomechanical measurements were conducted by M.K.M.H. and M.P. Digital densitometry measurements and analysis were done by R.S. M.K.M.H. wrote the manuscript with critical revision and contribution by all co‐authors. Finally, all the authors have approved the submitted version of the manuscript.
